# Thrombolysis implementation intervention and clinical outcome: a secondary analysis of a cluster randomized trial

**DOI:** 10.1186/s12872-020-01705-9

**Published:** 2020-10-06

**Authors:** Md Golam Hasnain, Christine L. Paul, John R. Attia, Annika Ryan, Erin Kerr, Christopher Oldmeadow, Catherine A. D’Este, Andrew Bivard, Isobel J. Hubbard, Abul Hasnat Milton, Christopher R. Levi

**Affiliations:** 1 School of Medicine and Public Health (SMPH), University of Newcastle (UoN), Callaghan, Australia; 2 Hunter Medical Research Institute (HMRI), New Lambton Heights, Australia; 3 John Hunter Hospital (JHH), New Lambton Heights, Australia; 4 National Centre for Epidemiology and Population Health, Research School of Population Health, Australian National University (ANU), Canberra, Australia; 5 Royal Melbourne Hospital, Parkville, Australia; 6 Epidemiology Resource Centre, Dhaka, Bangladesh; 7 The Sydney Partnership for Health, Education, Research & Enterprise (SPHERE), Sydney, Australia

**Keywords:** Ischemic stroke, Intravenous thrombolysis, Implementation intervention, Clinical outcome

## Abstract

**Background:**

Multiple studies have attempted to increase the rate of intravenous thrombolysis for ischemic stroke using interventions to promote adherence to guidelines. Still, many of them did not measure individual-level impact. This study aimed to make a posthoc comparison of the clinical outcomes of patients in the “Thrombolysis ImPlementation in Stroke (TIPS)” study, which aimed to improve rates of intravenous thrombolysis in Australia.

**Methods:**

A posthoc analysis was conducted using individual-level patient data. Excellent (Three-month post treatment modified Rankin Score 0–2) and poor clinical outcome (Three-month post treatment modified Rankin Score 5–6) and post treatment parenchymal haematoma were the three main outcomes, and a mixed logistic regression model was used to assess the difference between the intervention and control groups.

**Results:**

There was a non-significant higher odds of having an excellent clinical outcome of 57% (odds ratio: 1.57; 95% CI: 0.73–3.39) and 33% (odds ratio: 1.33; 95% CI: 0.73–2.44) during the active-and post-intervention period respectively, for the intervention compared to the control group. A non-significant lower odds of having a poor clinical outcome was also found in the intervention, relative to control group of 4% (odds ratio: 0.96; 95% CI: 0.56–2.07) and higher odds of having poor outcome of 44% (odds ratio: 1.44 95% CI: 0.61–3.41) during both active and post-intervention period respectively. Similarly, a non-significant lower odds of parenchymal haematoma was also found for the intervention group during the both active- (odds ratio: 0.53; 95% CI: 0.21–1.32) and post-intervention period (odds ratio: 0.96; 95% CI: 0.36–2.52).

**Conclusion:**

The TIPS multi-component implementation approach was not effective in reducing the odds of post-treatment severe disability at 90 days, or post-thrombolysis hemorrhage.

**Trial registration:**

Clinical Trial Registration-URL: http://www.anzctr.org.au/ Unique Identifier: ACTRN12613000939796.

## Background

Internationally and in Australia, stroke is a leading cause of death and disability [1, 2]. Improved outcomes after Acute Ischemic Stroke (AIS) can be achieved with intravenous thrombolysis administered within 4.5 h of symptom onset [3]. Despite being the guideline-recommended treatment option for AIS, the average rate of intravenous thrombolysis implementation was only 7% across Australia at the time this health systems trial was conducted [2] and much lower in regional hospitals [4]. Several pre and in-hospital barriers have been identified as contributing to the poor implementation of intravenous thrombolysis, including sub-optimal triage, lack of appropriate infrastructure and expertise, and physicians’ uncertainty in prescribing intravenous thrombolysis [5].

The Thrombolysis Implementation in Stroke (TIPS) study, was a cluster-randomized trial which aimed to improve thrombolysis rates through a multi-level, multi-component, in-hospital intervention which was implemented in 10 of 20 study hospitals across Australia [6]. The trial resulted in a transient uplift in intravenous thrombolysis rates in the intervention hospitals. However, the primary outcome of the trial was an overall non-significant increase in thrombolysis rates in the intervention versus control sites which was not sustained beyond the 16- month intervention period [7]. As described in the main trial outcome paper [7], there were challenges with intervention implementation at some regional hospitals, and the uptake of “best practice” knowledge translation strategies was limited by clinical leadership availability and staffing constraints [8]. However, despite the non-significant change in overall thrombolysis rates seen in the trial, it was considered possible that the TIPS intervention may have had a positive effect on individual patient clinical outcomes, for example, influencing improved selection of cases for thrombolysis treatment and streamlining of workflows [9]. A posthoc analysis of the TIPS database provides a unique opportunity to explore whether differences in individual patient clinical outcomes occurred within and between study groups.

This posthoc analysis of the TIPS data tested the hypothesis that the three-month clinical outcomes of disability (measured through modified Rankin Scale, mRS) and post-thrombolysis hemorrhage would show more favourable profiles in patients managed at the hospitals exposed to the TIPS intervention, relative to control hospitals.

## Methods

TIPS was a clustered randomized controlled trial that involved 20 hospitals from three Australian states: Victoria, New South Wales, and Queensland. All hospitals that participated in the TIPS study had either a Stroke Care Unit or staffing equivalent to a stroke physician and a nurse, and an emergency department. Ethical approval for the TIPS study was obtained from relevant human research ethics committees in each state, from each participating hospital, and The University of Newcastle Human Research Ethics Committee. The study adheres to CONSORT guidelines (Supplement 1).

Hospitals were randomized, stratified by baseline intravenous thrombolysis rate, either to receive a multi-component multi-disciplinary collaborative intervention, which focused on the safe use of intravenous thrombolysis therapy for AIS patients; or to continue with standard care. Blinding was not possible because of the involvement of staff in the intervention activities. Pre-intervention data were collected for each hospital for 12–24 months before implementation of the intervention. Following the pre-intervention period, a 16-month active intervention period occurred during which intervention hospitals received the intervention while control hospitals continued with standard care. The intervention was then withdrawn, and outcomes monitored during a 12-month post-intervention period.

### Measures

Data on all thrombolysed cases were entered into a TIPS study-specific database in a de-identified form by the hospital staff. The variables of interest were pre- and three-month post-thrombolysis mRS and rates of post-thrombolysis parenchymal haematoma as detected in routine clinical practice following guideline based post-thrombolysis imaging recommendations [10]. Hospital staff entering data were trained in the Computed Tomography (CT) and Magnetic Resonance Imaging (MRI) characteristics of the European Cooperative Acute Stroke Study 2 (ECASS 2) classification system and were asked to review source imaging in the classification of haemorrhagic transformation [11]. The TIPS study-specific database included questions and an algorithm to ensure consistency in the classification of the mRS. At 90 days post-admission, patients were contacted by hospital staff to record the mRS either by phone or in a clinic. We used two dichotomous definitions of the 90-day clinical mRS outcome to reflect the most favourable and most catastrophic outcomes: excellent clinical outcome (mRS 0–1 vs 2–6), and poor outcomes (mRS 5–6 vs. 0–4). Patients who were thrombolysed also had their baseline, and where available follow-up, imaging recorded; all patients received a baseline non-contrast CT at a minimum. Hospital staff for the presence of a hemorrhage using the ECASS 2 scoring system assessed all 24-h imaging [11].

Haemorrhagic events were classified according to clinical and CT criteria. Haemorrhagic infarction 1 (HI1) was defined as small petechiae along the margins of the infarct; haemorrhagic infarction 2 (HI2) as confluent petechiae within the infarcted area but no space-occupying effect; parenchymal haemorrhage (PH1) defined as blood clots in 30% or less of the infarcted area with some slight space-occupying effect; and parenchymal haemorrhage (PH2) defined as blood clots in more than 30% of the infarcted area with substantial space-occupying effect [11]. For our study, we defined both PH1 and PH2 as post-treatment parenchymal haematoma (PH).

Other data of interest included age, gender, pre-stroke mRS, pre- and post-thrombolysis National Institutes of Health Stroke Scale (NIHSS) which measures the severity of stroke; and pre-thrombolysis systolic blood pressure (SBP) on admission which is a risk factor for poor outcome.

### Intervention

**Fig. 1:**
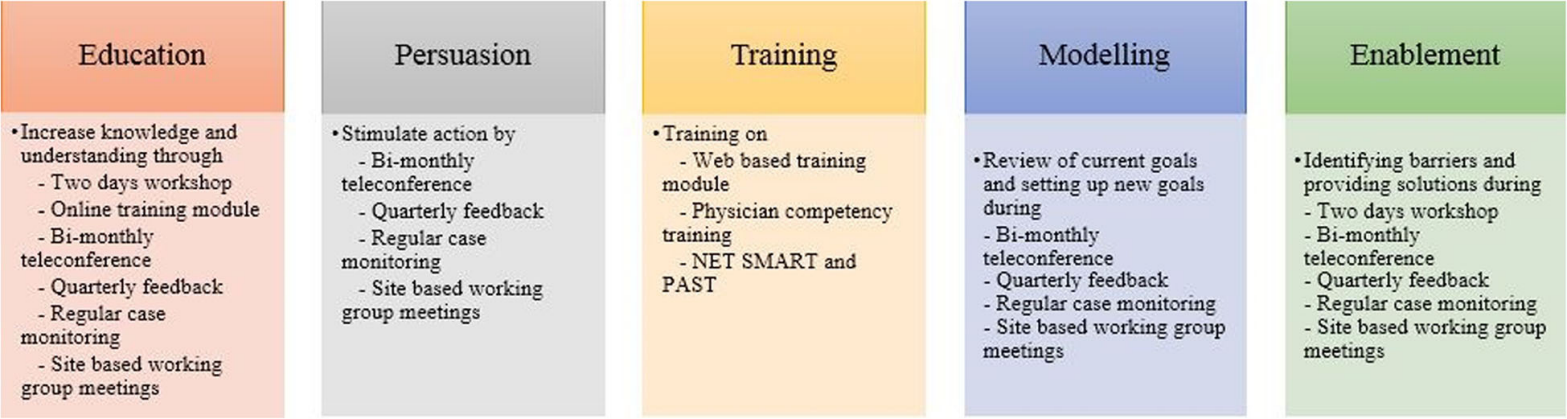
Legend: Framework of interventional activity

The intervention was a multi-component, collaborative intervention that was based on a knowledge translation approach and the behavioural change wheel [12] (Fig. 1). The intervention sites signed a written collaborative agreement and then participated in a site-specific situational analysis which was followed by a collaborative workshop, teleconferences, feedback, and monitoring as detailed in Fig. 1. The control sites were not provided with any intervention and could be considered a ‘usual care’ condition.

### Statistical analysis

The main study was powered to detect a difference in thrombolysis rates between the two groups [6]. Assuming the same parameters as the original power calculation (cluster coefficient of variation = 0.4; alpha = 0.05; 10 clusters per arm), this secondary analysis of all thrombolysed cases (*n* = 1559) had 80% power to detect absolute differences in these key secondary outcomes between intervention and control groups of between 22 and 33% for outcomes with prevalence’s ranging from 50 to 75%. The analysis population is all those that were treated with intravenous thrombolysis. Data was not available on patients that were considered for intravenous thrombolysis but not thrombolysed. Primary analyses compared outcomes between intervention and control group at the active intervention and post-intervention phases. Three mixed effects logistic regression models were used to assess the difference in the study outcomes, proportion of patients with excellent and poor clinical outcome and with PH, between the intervention and control arm during the both active and post intervention period separately. These three models included fixed effects for baseline thrombolysis rates (site-level), pre-morbid mRS, baseline NIHSS; treatment group. Another three mixed-effects logistic regression models were used to determine the effect of the educational intervention on changes in clinical outcomes from pre to active and pre to post-intervention period. These three models included fixed effects for baseline thrombolysis rates (site-level), pre-morbid mRS, baseline NIHSS; treatment group, period (pre vs post) and the interaction between treatment and period. All the above mentioned models included a random intercept for hospital site to account for correlations of individuals within the same site. Due to missing data on mRS outcome, multiple imputation analyses using the chained regression equations method were performed. The missing data was imputed based on hospital site, pre-morbid mRS, NIHSS, age and gender. The imputation process (*n* = 200 imputations) was conducted assuming the data were missing at random and combined using Rubin’s method. Statistical significance was defined as a two-tailed *p*-value of < 0.05. Statistical analyses were programmed using SAS v9.4 (SAS Institute, Cary, North Carolina, USA).

## Results

**Fig. 2:**
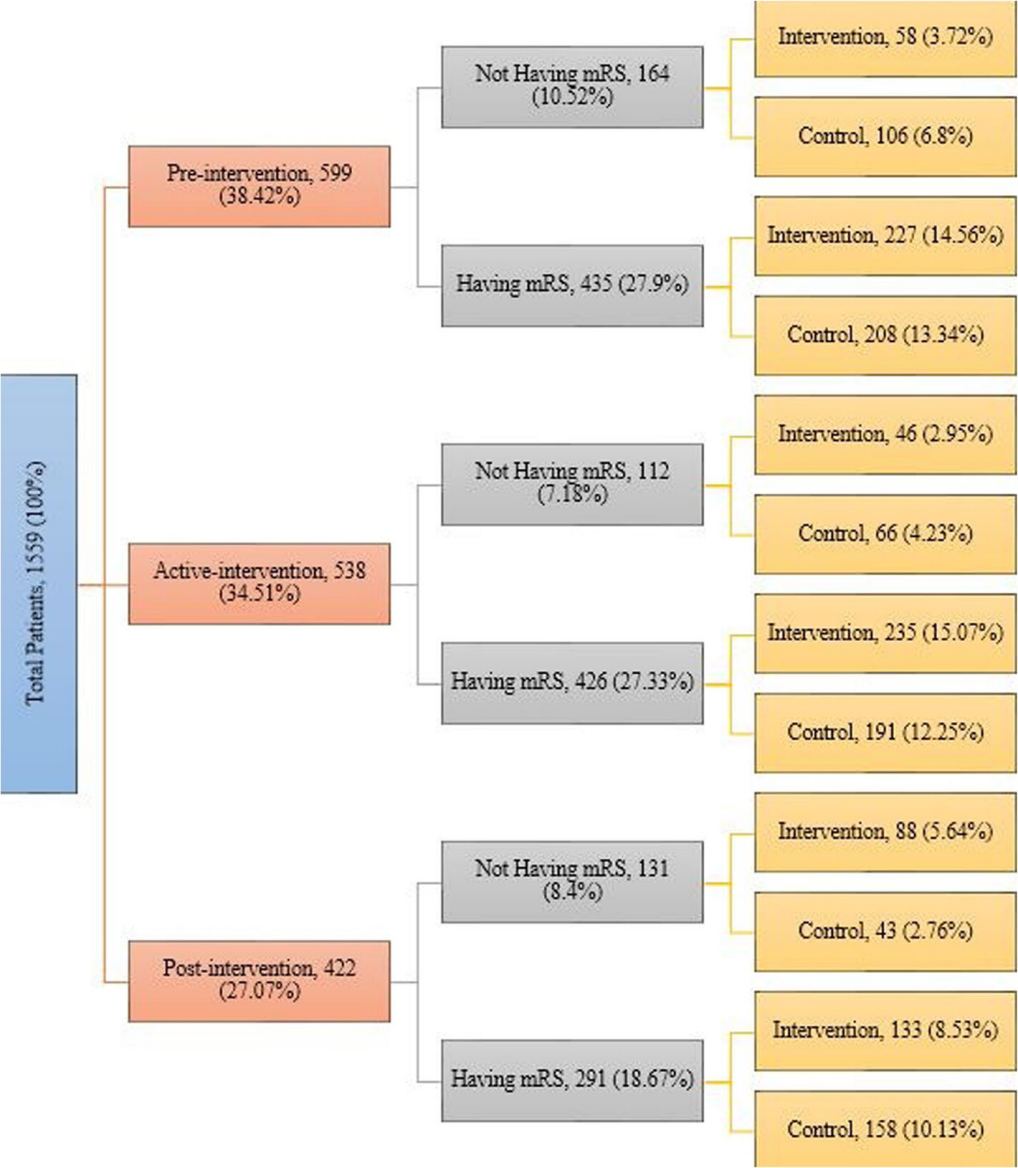
Legend: Distribution of patients within two study arms over three study periods

From January 2011 to December 2015, data on 1559 patients who received intravenous thrombolysis were recorded from the 20 hospitals that participated in the TIPS study. This included pre-intervention period data from 599 (38%) patients, active-intervention period data from 538 (35%) patients and post-intervention period data from 422 (27%) patients. Finally, a total of 1184 (76%) patient’s data were included. The pattern of missing data for 3 month post treatment mRS between intervention and control groups is reported in Fig. 2. The pattern of missing values related to patient characteristics has been described in Supplement 2. Overall, the mean (Standard Deviation; SD) age was 71.34 (14.55) years, and 533 (54%) were male; 70% (*n* = 393) of patients reported no pre-stroke disability (mRS = 0) during the pre-intervention phase, with the corresponding proportions being 64% (*n* = 318) in the active-intervention period and 55% (*n* = 188) in the post-intervention period. A detailed description of patient characteristics has shown in Table 1. No differences in the patient selection were observed among intervention and control hospitals at each time point. A table comparing the clinical and demographic features of patients with vs without mRS at 90 days has been added in Supplement 3.

**Table 1:** Description of patient characteristics based on intervention and intervention implementation phase

	Pre-Intervention		Active Intervention		Post-Intervention	
	Intervention	Control	Intervention	Control	Intervention	Control
Age in years						
- Mean ± SD	71.78 ± 14.22	70.37 ± 13.81	72.47 ± 6.55	71.09 ± 13.31	73 ± 15.22	70.66 ± 15.41
Gender, n (%)						
- Female	135 (47)	138 (44)	152 (54)	125 (49)	90 (48)	92 (46)
- Male	150 (53)	176 (56)	128 (46)	132 (51)	98 (52)	109 (54)
Systolic Blood Pressure in mm of Hg						
- Mean ± SD	151.56 ± 24.60	148.93 ± 23.88	147.20 ± 24.16	148.83 ± 24.06	146.51 ± 24.84	150.85 ± 23.95
Diastolic Blood Pressure in mm of Hg						
- Mean ± SD	84.20 ± 16.05	79.73 ± 14.34	83.57 ± 16.27	78.03 ± 13.41	86.06 ± 16.29	80.04 ± 13.93
History of Hypertension, n (%)						
- No	101 (36)	101 (34)	90 (35)	70 (27)	63 (39)	64 (32)
- Yes	179 (64)	196 (66)	164 (65)	187 (73)	98 (61)	134 (68)
History of Diabetes, n (%)						
- No	224 (81)	235 (78)	206 (80)	192 (77)	124 (77)	151 (76)
- Yes	51 (19)	67 (22)	50 (20)	65 (23)	37 (23)	48 (24)
History of Previous Stroke, n (%)						
- No	243 (87)	246 (83)	211 (85)	213 (85)	132 (81)	161 (84)
- Yes	35 (13)	51 (17)	37 (15)	39 (15)	31 (19)	30 (16)
History of Atrial Fibrillation, n (%)						
- No	167 (61)	213 (70)	165 (67)	158 (63)	114 (73)	143 (72)
- Yes	107 (39)	92 (30)	82 (33)	92 (37)	42 (27)	55 (28)
Pre-morbid mRS, n (%)						
- mRS 0–2	231 (88)	279 (93)	205 (83)	223 (89)	110 (76)	172 (88)
- mRS 3–4	26 (10)	18 (6)	43 (17)	27 (10.5)	33 (23)	23 (11.5)
- mRS 5	4 (2)	2 (1)	0 (0)	1 (0.5)	2 (1)	1 (0.5)
Baseline NIHSS						
- Mean ± SD	11.71 ± 6.99	11.50 ± 6.51	10.05 ± 6.75	10.77 ± 6.39	10.53 ± 6.65	11.85 ± 6.78

### Disability outcomes (mRS)

There was a non-significantly higher odds of having an excellent clinical outcome of 57% (odds ratio: 1.57; 95% CI: 0.73–3.39; Table 2) during the active intervention period and 33% (odds ratio: 1.33; 95% CI: 0.73–2.44) during the post-intervention period. The intervention was associated with a non-significantly lower odds of having a poor clinical outcome of 4% (odds ratio: 0.96; 95% CI: 0.56–2.07;) during the active intervention period and a higher odds of 44% (odds ratio: 1.44; 95% CI: 0.61–3.41;) during the post-intervention period, compared to the control group. The within-group analysis compared to baseline and the comparison between the intervention and control arm for both active and post-intervention period are shown in Table 3, and the effects remained non- significant. The disability outcome result also remained non-significant, even after applying the multiple imputation method (Supplement 4).

**Table 2:** Odds ratio (OR) with 95% Confidence Interval (CI) between intervention vs control during active and post intervention period

	Active Intervention Period			Post Intervention Period		
	Number, n (%)	OR (95% CI)	***p***-value	Number, n (%)	OR (95% CI)	***p***-value
**Excellent Outcome (Three month post treatment mRS 0–2)**						
Control	74 (39%)	Reference	Reference	57 (36%)	Reference	Reference
Intervention	106 (45%)	1.57 (0.73–3.39)	0.250	72 (44%)	1.33 (0.73–2.44)	0.357
**Poor Outcome (Three month post treatment mRS 5–6)**						
Control	29 (15%)	Reference	Reference	22 (14%)	Reference	Reference
Intervention	34 (14%)	0.96 (0.56–2.07)	0.817	24 (15%)	1.44 (0.61–3.41)	0.405
**PH (Post treatment)**						
Control	16 (6.2%)	Reference	Reference	12 (6%)	Reference	Reference
Intervention	9 (3.2%)	0.53 (0.21–1.32)	0.173	10 (4.5%)	0.96 (0.36–2.52)	0.928

Mixed effects logistic mixed model was used

Models were controlled for baseline thrombolysis rate, pre-morbid modified Rankin Score (mRS) and baseline National Institute of Health Stroke Scale (NIHSS)

A *p*-value < 0.05 was considered as significant

**Table 3:** Within group and between group change between pre vs. Active and pre vs. post intervention period for the both intervention and control

	Within Group Change						Between Group Change	
	Intervention			Control			Intervention vs. Control	
	Number, n (%)	OR (95% CI)	***p***-value	Number, n (%)	OR (95% CI)	***p***-value	OR (95% CI)	***p***-value
**Excellent Outcome (Three month post treatment mRS 0–2)**								
Pre-intervention period	107 (47%)	Reference	Reference	66 (32%)	Reference	Reference	Reference	Reference
Active intervention period	106 (45%)	0.97 (0.62–1.51)	0.878	74 (39%)	1.15 (0.60–1.51)	0.834	0.98 (0.54–1.92)	0.966
Post intervention period	72 (44%)	0.96 (0.56–1.65)	0.892	57 (36%)	1.07 (0.59–1.59)	0.906	0.99 (0.48–2.05)	0.983
**Poor Outcome (Three month post treatment mRS 5–6)**								
Pre-intervention period	37 (16%)	Reference	Reference	44 (21%)	Reference	Reference	Reference	Reference
Active intervention period	34 (14%)	0.84 (0.47–1.51)	0.568	29 (15%)	0.78 (0.43–1.40)	0.397	1.09 (0.48–2.49)	0.843
Post intervention period	24 (15%)	0.43 (0.19–0.95)	0.047	22 (14%)	0.58 (0.31–1.08)	0.087	0.74 (0.27–2.03)	0.559
**PH (Post treatment)**								
Pre-intervention period	21 (7.4%)	Reference	Reference	22 (7%)	Reference	Reference	Reference	Reference
Active intervention period	9 (3.2%)	0.51 (0.22–1.18)	0.115	16 (6.2%)	0.95 (0.47–1.90)	0.880	0.54 (0.18–1.60)	0.265
Post intervention period	10 (4.5%)	0.82 (0.35–1.95)	0.880	12 (6%)	0.90 (0.42–1.91)	0.780	0.92 (0.29–2.87)	0.884

Mixed effects logistic mixed model was used

Models were controlled for baseline thrombolysis rate, pre-morbid modified Rankin Score (mRS) and baseline National Institute of Health Stroke Scale (NIHSS)

A *p*-value < 0.05 was considered as significant

Within Group Change: It shows only the change from pre- to active intervention period and pre- to post intervention period for both the intervention and control arm separately

Between Group Change: It shows the difference between the within group changes of the intervention and control arm for both the active and post intervention period separately

### Parenchymal Haematoma (PH)

During active and post-intervention period, the intervention group showed a non-significant decrease in the odds of having post-treatment PH by 47% (OR: 0.53, 95% CI: 0.21–1.32) and 4% (OR: 0.96, 95% CI: 0.36–2.52), during active and post-intervention period respectively (Table 2).

The within-group analysis compared to baseline and the comparison between the intervention and control arm for both active and post-intervention period are shown in Table 3, and the effects remained non- significant. The outcome result was also non-significant after applying the multiple imputation method to explore the impact of various assumptions regarding the missing data (Supplement 5).

## Discussion

Although the TIPS trial did observe a significant but transient increase in thrombolysis rates (OR = 1·6; 95% CI; 1·1–2·3) during the initial 16-month active intervention period [7], this posthoc analysis at the individual patient level, found that the intervention promoting thrombolysis implementation did not result in any significant difference in excellent or poor outcome between patients treated at intervention versus control hospitals. A non-significant decrease in the rate of hemorrhages following intravenous thrombolysis was seen during both active and post-intervention period. Therefore, our hypothesis that exposure to the TIPS intervention may have resulted in enhanced care and more favourable outcomes for thrombolysis patients treated in the intervention hospitals is not supported by the data. This lack of influence on individual patient clinical outcomes may be due to several factors. The first may be purely related to the size of the available sample and available power to detect a statistically significant change. Our point estimates indicate a possible increase in excellent outcomes and a potential decrease in poor outcomes and haemorrhages. However, despite an efficient statistical analytical method using baseline data, these proportions did not reach statistical significance. Of course, the main trial was designed to identify a change in the implementation of best evidence practice (i.e. the proportion of stroke cases receiving thrombolysis) rather than a change in clinical outcome.

A second factor is the trend throughout the trial for increasingly favourable outcome rates in the control group. The control group demonstrated an increase in the proportion of patients with excellent outcomes in both the active and post-intervention periods. Such results could have several explanations. In Australia, from 2010, several national-level health policy initiatives were released with the intent to improve the management of stroke broadly including intravenous thrombolysis [13]. The revised Clinical Guidelines for Stroke Management were released in 2010 by the NHMRC. The National Stroke Audit Reports for Acute Services (2011, 2013, 2015 and 2017) were delivered to the health professionals through online health portals [13]. A new Clinical Council was established by the National Stroke Foundation in 2011 to improve the quality of stroke care by ensuring that physicians were aware of the latest clinical evidence and initiatives relating to quality stroke care [13]. These programs and initiatives focused were active during the trial period and could have driven the secular trends observed in the outcomes of interest. It is also conceivable that there was some degree of contamination and some crossover of interventional material between control and intervention sites, despite all care being taken to avoid this occurrence, due to the relatively small and tight-knit community of stroke care providers in Australia.

Further interpretation of the negative results of this individual patient analysis aligns with the well-recognised difficulty in shifting clinician behaviour and the neutral outcome of the main trial, where a comprehensive and multifaceted package of support and education aimed at knowledge translation failed to substantially or sustained oblique change practice. This difficulty is mirrored in several previous studies such as the PRomoting ACute Thrombolysis in Ischemic StrokE (PRACTISE) trial. PRACTICE was a cluster randomised trial in The Netherlands which used the breakthrough approach aiming to improve the thrombolysis rates. PRACTISE intervention failed to achieve a significant overall increase in thrombolysis rates. The study also evaluated the rate of symptomatic intracranial bleeding complications, the good clinical outcome at 3 months (mRS < 3) and mortality, and the effect of PRACTICE intervention on clinical outcome was non-significant. Finally, the study concluded that unregistered comorbidities and risk factors might have contaminated the findings [14]. The INcreasing Stroke Treatment through INterventional Change Tactics (INSTINCT) cluster randomised controlled trial in the US used a multilevel, barrier assessment-interactive educational intervention [15]. This trial also showed non-significant between-group differences in thrombolysis rates [15]. It highlighted the difficulties of knowledge translational strategies due to local factors such as familiarity with and motivation to adhere to the guidelines [15]. However, the study did not perform any individual patient-level analysis [15]. Currently, therefore, to our knowledge, there is no clinical trial evidence to suggest that health systems interventions targeting implementation of best evidence practise in stroke thrombolysis can improve thrombolysis patients’ clinical outcomes.

It should also note that there is also uncertainty around the choice of clinical outcome of relevance in health systems trials such as TIPS. The limitations of the modified Rankin score are well recognised [16], and it is possible that other patient-reported outcome measures such as quality of life scores would be more relevant secondary outcome measures for trials of this type.

## Conclusion

The TIPS multi-component implementation approach was not effective in improving the odds of the 90-day excellent outcome or reducing the odds of having 90 days post-treatment poor outcome and severe post-treatment haemorrhage. Further study of the clinical impact of efforts to improve thrombolysis rate is warranted.

## Supplementary information

**Additional file 1 **: **Supplement 1:** CONSORT 2010 checklist of information to include when reporting a randomised trial**. Supplement 2:** Number and percentage of missing values, n (%), for each patient characteristics. **Supplement 3:** Table comparing the clinical and demographic features of patients with vs without mRS at 90 day. **Supplement 4:** Odds ratio after multiple imputation between intervention vs control during active and post intervention period. **Supplement 5:** Within group and between group change after multiple imputation between pre vs. Active and pre vs. post intervention period for the both intervention and control.

## Data Availability

The datasets used and/or analysed during the current study are available from the corresponding author on reasonable request.

## Abbreviations

AIS: Acute ischemic stroke
CT: Computed tomography
ECASS: European cooperative acute stroke study
HI: Haemorrhagic infarction
INSTINCT: INcreasing stroke treatment through INterventional change tactics
MRI: Magnetic resonance imaging
mRS: Modified Rankin scale
NIHSS: National Institute of health stroke scale
PH: Parenchymal Haematoma
PRACTISE: PRomoting ACute thrombolysis in ischemic StrokE
SBP: Systolic blood pressure
