# Supplementary material for: Thrombolysis implementation intervention and clinical outcome: a secondary analysis of a cluster randomized trial
Source: BMC Cardiovasc Disord. 2020 Oct 6;20:432. doi: 10.1186/s12872-020-01705-9 (PMC7542125; doi:10.1186/s12872-020-01705-9)
Supplement: Supplementary file 1 — Additional file 1 : Supplement 1: CONSORT 2010 checklist of information to include when reporting a randomised trial. Supplement 2: Number and percentage of missing values, n (%), for each patient characteristics. Supplement 3: Table comparing the clinical and demographic features of patients with vs without mRS at 90 day. Supplement 4: Odds ratio after multiple imputation between intervention vs control during active and post intervention period. Supplement 5: Within group and between group change after multiple imputation between pre vs. Active and pre vs. post intervention period for the both intervention and control. [file 12872_2020_1705_MOESM1_ESM.docx]

Supplement 1: CONSORT 2010 checklist of information to include when reporting a randomised trial

| Section/Topic | Item No | Checklist item | Reported on page No |
| --- | --- | --- | --- |
| Title and abstract | | | |
|  | 1a | Identification as a randomised trial in the title | Paragraph 1; Page 1 |
|  | 1b | Structured summary of trial design, methods, results, and conclusions (for specific guidance see CONSORT for abstracts) | Abstract; Page 4-5 |
| Introduction | | | |
| Background and objectives | 2a | Scientific background and explanation of rationale | Background; Page 6-7 |
|  | 2b | Specific objectives or hypotheses | Background; Paragraph 1; Page 7 |
| Methods | | | |
| Trial design | 3a | Description of trial design (such as parallel, factorial) including allocation ratio | Methods; Paragraph 1 Page 7 |
|  | 3b | Important changes to methods after trial commencement (such as eligibility criteria), with reasons | NA |
| Participants | 4a | Eligibility criteria for participants | Methods; Paragraph 1 Page 7 |
|  | 4b | Settings and locations where the data were collected | Methods; Paragraph 2 Page 7 |
| Interventions | 5 | The interventions for each group with sufficient details to allow replication, including how and when they were actually administered | Methods; Paragraph 1 Page 7 and Intervention Page 9 |
| Outcomes | 6a | Completely defined pre-specified primary and secondary outcome measures, including how and when they were assessed | Measures; Page 8-9 |
|  | 6b | Any changes to trial outcomes after the trial commenced, with reasons | NA |
| Sample size | 7a | How sample size was determined | NA |
|  | 7b | When applicable, explanation of any interim analyses and stopping guidelines | NA |
| Randomisation: |  |  |  |
| Sequence generation | 8a | Method used to generate the random allocation sequence | NA |
|  | 8b | Type of randomisation; details of any restriction (such as blocking and block size) | NA |
| Allocation concealment mechanism | 9 | Mechanism used to implement the random allocation sequence (such as sequentially numbered containers), describing any steps taken to conceal the sequence until interventions were assigned | NA |
| Implementation | 10 | Who generated the random allocation sequence, who enrolled participants, and who assigned participants to interventions | NA |
| Blinding | 11a | If done, who was blinded after assignment to interventions (for example, participants, care providers, those assessing outcomes) and how | NA |
|  | 11b | If relevant, description of the similarity of interventions | NA |
| Statistical methods | 12a | Statistical methods used to compare groups for primary and secondary outcomes | Statistical Analysis; Page 9-10 |
|  | 12b | Methods for additional analyses, such as subgroup analyses and adjusted analyses | Statistical Analysis; Page 9-10 |
| Results | | | |
| Participant flow (a diagram is strongly recommended) | 13a | For each group, the numbers of participants who were randomly assigned, received intended treatment, and were analysed for the primary outcome | Figure 2 |
|  | 13b | For each group, losses and exclusions after randomisation, together with reasons | NA |
| Recruitment | 14a | Dates defining the periods of recruitment and follow-up | Results; Page 10 |
|  | 14b | Why the trial ended or was stopped | NA |
| Baseline data | 15 | A table showing baseline demographic and clinical characteristics for each group | Supplement 2 |
| Numbers analysed | 16 | For each group, number of participants (denominator) included in each analysis and whether the analysis was by original assigned groups | Paragraph 1; Page 10 |
| Outcomes and estimation | 17a | For each primary and secondary outcome, results for each group, and the estimated effect size and its precision (such as 95% confidence interval) | Disability Outcomes and Parenchymal Haematoma; Page 11-12 |
|  | 17b | For binary outcomes, presentation of both absolute and relative effect sizes is recommended | Disability Outcomes and Parenchymal Haematoma; Page 11-12 |
| Ancillary analyses | 18 | Results of any other analyses performed, including subgroup analyses and adjusted analyses, distinguishing pre-specified from exploratory | NA |
| Harms | 19 | All important harms or unintended effects in each group (for specific guidance see CONSORT for harms) | NA |
| Discussion | | | |
| Limitations | 20 | Trial limitations, addressing sources of potential bias, imprecision, and, if relevant, multiplicity of analyses | Paragraph 1-2; Page 13-14 |
| Generalisability | 21 | Generalisability (external validity, applicability) of the trial findings | Paragraph 2; Page 14 |
| Interpretation | 22 | Interpretation consistent with results, balancing benefits and harms, and considering other relevant evidence | Discussion; Paragraph 2; Page 12 |
| Other information | | |  |
| Registration | 23 | Registration number and name of trial registry | Trial Registration; Page 5 |
| Protocol | 24 | Where the full trial protocol can be accessed, if available | Reference 6; Page 17 |
| Funding | 25 | Sources of funding and other support (such as supply of drugs), role of funders | Funding; Page 16 |

**Supplement 2:** Number and percentage of missing values, n (%), for each patient characteristics.

|  | Pre-Intervention | | Active Intervention | | Post-Intervention | |
| --- | --- | --- | --- | --- | --- | --- |
|  | Intervention | Control | Intervention | Control | Intervention | Control |
| Age | 18 (6) | 24 (8) | 19 (7) | 15 (6) | 43 (19) | 8 (4) |
| Gender | 0 (0) | 0 (0) | 1 (0.4) | 0 (0) | 33 (15) | 0 (0) |
| Systolic Blood Pressure | 0 (0) | 1 (0.3) | 0 (0) | 0 (0) | 33 (15) | 0 (0) |
| Diastolic Blood Pressure | 0 (0) | 1 (0.3) | 0 (0) | 0 (0) | 33 (15) | 0 (0) |
| History of Hypertension | 5 (2) | 17 (5) | 27 (10) | 0 (0) | 60 (27) | 3 (1) |
| History of Diabetes | 10 (4) | 12 (4) | 25 (9) | 0 (0) | 60 (27) | 2 (1) |
| History of Previous Stroke | 7 (2) | 17 (5) | 33 (12) | 5 (2) | 58 (26) | 10 (5) |
| History of Atrial Fibrillation | 11 (4) | 9 (3) | 34 (12) | 7 (3) | 65 (29) | 3 (1) |
| Pre-morbid mRS | 0 (0) | 15 (5) | 33 (12) | 6 (2) | 77 (35) | 5 (2) |
| Baseline NIHSS | 0 (0) | 8 (3) | 1 (0.4) | 5 (2) | 37 (17) | 13 (6) |

**Supplement 3:** Table comparing the clinical and demographic features of patients with vs without mRS at 90 day

| Three-Month Post Thrombolysis mRS Data | Pre-Intervention | | | | Active Intervention | | | | Post-Intervention | | | |
| --- | --- | --- | --- | --- | --- | --- | --- | --- | --- | --- | --- | --- |
|  | Intervention | | Control | | Intervention | | Control | | Intervention | | Control | |
|  | *Non-Missing* | *Missing* | *Non-Missing* | *Missing* | *Non-Missing* | *Missing* | *Non-Missing* | *Missing* | *Non-Missing* | *Missing* | *Non-Missing* | *Missing* |
| Age in Years  (Mean ± SD) | 72.06 (14.32) | 67.07 (11.87) | 70.46 (13.42) | 70.10 (14.91) | 72.66 (16.98) | 70.43 (11.19) | 71.06 (13.34) | 71.23 (13.33) | 73.86 (14.40) | 69.49 (18.03) | 71.18 (15.60) | 67.8 (14.23) |
| Female Gender, n (%) | 129 (48) | 6 (40) | 93 (40) | 45 (56) | 138 (54) | 120 (47) | 105 (48) | 20 (50) | 64 (35) | 26 (65) | 79 (46) | 13 (42) |
| Systolic Blood Pressure in mm of Hg  (Mean ± SD) | 151.01 (24.73) | 161.47 (20.24) | 149.77 (22.86) | 146.51 (26.58) | 148.23 (23.83) | 135.70 (25.41) | 148.83 (24.40) | 148.83 (22.41) | 150.39 (23.42) | 131.67 (24.80) | 150.83 (23.24) | 150.94 (28.00) |
| Diastolic Blood Pressure in mm of Hg  (Mean ± SD) | 83.91 (16.12) | 89.53 (14.32) | 79.59 (14.69) | 80.15 (13.40) | 82.49 (15.51) | 95.74 (19.70) | 78.23 (13.09) | 76.98 (15.18) | 84.48 (15.45) | 92.13 (18.13) | 80.45 (13.98) | 77.77 (13.65) |
| History of Hypertension, n (%) | 171 (63) | 8 (53) | 145 (62) | 51 (63) | 152 (59) | 12 (53) | 158 (73) | 29 (73) | 90 (50) | 8 (20) | 115 (68) | 19 (61) |
| History of Diabetes, n (%) | 49 (18) | 2 (13) | 51 (22) | 16 (20) | 49 (19) | 1 (4) | 54 (25) | 11 (28) | 32 (18) | 12 (13) | 39 (23) | 9 (29) |
| History of Previous Stroke, n (%) | 35 (13) | 00 (00) | 39 (17) | 12 (15) | 36 (14) | 1 (4) | 30 (14) | 9 (23) | 32 (18) | 2 (5) | 25 (15) | 4 (13) |
| History of Atrial Fibrillation, n (%) | 101 (37) | 6 (40) | 73 (31) | 19 (23) | 81 (32) | 1 (4) | 81 (37) | 11 (28) | 38 (21) | 4 (10) | 54 (32) | 1 (3) |

**Supplement 4:** Odds ratio after multiple imputation between intervention vs control during active and post intervention period.

|  | **Active Intervention Period** | | | **Post Intervention Period** | | |
| --- | --- | --- | --- | --- | --- | --- |
|  | **Number, n (%)** | **OR (95% CI)** | **p-value** | **Number, n (%)** | **OR (95% CI)** | **p-value** |
| **Excellent Outcome (Three month post treatment mRS 0-2)** |  |  |  |  |  |  |
| Control | 74 (39%) | Reference | Reference | 57 (36%) | Reference | Reference |
| Intervention | 106 (45%) | 1.04 (0.59-1.83) | 0.9001 | 72 (44%) | 1.82 (0.78-4.21) | 0.1636 |
| **Poor Outcome (Three month post treatment mRS 5-6)** |  | | | | | |
| Control | 29 (15%) | Reference | Reference | 22 (14%) | Reference | Reference |
| Intervention | 34 (14%) | 1.13 (0.61-2.06) | 0.7004 | 24 (15%) | 0.99 (0.42-2.33) | 0.9745 |
| **PH (Post treatment)** |  | | | | | |
| Control | 16 (6.2%) | Reference | Reference | 12 (6%) | Reference | Reference |
| Intervention | 9 (3.2%) | 0.56 (0.23-1.36) | 0.1982 | 10 (4.5%) | 0.75 (0.29-1.92) | 0.5462 |

*Mixed effects logistic mixed model was used.

**Models were controlled for baseline thrombolysis rate, pre-morbid modified Rankin Score (mRS) and baseline National Institute of Health Stroke Scale (NIHSS).

***A p-value <0.05 was considered as significant.

**Supplement 5:** Within group and between group change after multiple imputation between pre vs. Active and pre vs. post intervention period for the both intervention and control.

|  | **Within Group Change** | | | | | | **Between Group Change** | |
| --- | --- | --- | --- | --- | --- | --- | --- | --- |
|  | **Intervention** | | | **Control** | | | **Intervention vs. Control** | |
|  | **Number, n (%)** | **OR (95% CI)** | **p-value** | **Number, n (%)** | **OR(95% CI)** | **p-value** | **OR(95% CI)** | **p-value** |
| **Excellent Outcome (Three month post treatment mRS 0-2)** |  | | | | | | | |
| Pre-intervention period | 107 (47%) | Reference | Reference | 66 (32%) | Reference | Reference | Reference | Reference |
| Active intervention period | 106 (45%) | 0.89 (0.61-1.29) | 0.5453 | 74 (39%) | 1.35 (0.89-2.06) | 0.1566 | 0.66 (0.38-1.16) | 0.1448 |
| Post intervention period | 72 (44%) | 1.23 (0.79-1.91) | 0.3517 | 57 (36%) | 1.34 (0.85-2.10) | 0.2059 | 0.92 (0.49-1.73) | 0.7966 |
| **Poor Outcome (Three month post treatment mRS 5-6)** |  | | | | | |  |  |
| Pre-intervention period | 37 (16%) | Reference | Reference | 44 (21%) | Reference | Reference | Reference | Reference |
| Active intervention period | 34 (14%) | 0.89 (0.53-1.49) | 0.6551 | 29 (15%) | 0.64 (0.38-1.08) | 0.0943 | 1.39 (0.67-2.90) | 0.3753 |
| Post intervention period | 24 (15%) | 1.34 (0.75-2.40) | 0.3711 | 22 (14%) | 1.70 (0.97-3.00) | 0.1527 | 0.79 (0.35-1.77) | 0.6762 |
| **PH (Post treatment)** |  | | | | | |  |  |
| Pre-intervention period | 21 (7.4%) | Reference | Reference | 22 (7%) | Reference | Reference | Reference | Reference |
| Active intervention period | 9 (3.2%) | 0.44 (0.20-0.98) | 0.0452 | 16 (6.2%) | 0.84 (0.43-1.65) | 0.6194 | 0.52 (0.18-1.48) | 0.2221 |
| Post intervention period | 10 (4.5%) | 0.56 (0.26-1.23) | 0.5462 | 12 (6%) | 0.82 (0.39-1.70) | 0.5890 | 0.69 (0.24-2.01) | 0.4960 |

*Mixed effects logistic mixed model was used.

**Models were controlled for baseline thrombolysis rate, pre-morbid modified Rankin Score (mRS) and baseline National Institute of Health Stroke Scale (NIHSS).

***A p-value <0.05 was considered as significant.

****Within Group Change: It shows only the change from pre- to active intervention period and pre- to post intervention period for both the intervention and control arm separately.

*****Between Group Change: It shows the difference between the within group changes of the intervention and control arm for both the active and post intervention period separately.
